# Coronary Artery Revascularization Evaluation—A Multicenter Registry With Seven Years of Follow‐Up

**DOI:** 10.1161/JAHA.113.000162

**Published:** 2013-04-24

**Authors:** Paul Kurlansky, Morley Herbert, Syma Prince, Michael J. Mack

**Affiliations:** 1 Florida Heart Research Institute, Miami, FL (P.K.); 2 Medical City Dallas Hospital, Dallas, TX (M.H.); 3 Cardiopulmonary Research Science and Technology Institute, Dallas, TX (S.P.); 4 Baylor Health Care System, The Heart Hospital Baylor Plano, Plano, TX (M.J.M.)

**Keywords:** angioplasty, coronary disease, follow‐up studies, revascularization, surgery

## Abstract

**Background:**

Data from randomized clinical trials comparing coronary artery bypass grafting (CABG) and percutaneous coronary intervention (PCI) may not accurately reflect current clinical practice, in which there is off‐label usage of drug‐eluting stents (DES). We undertook a prospective registry of coronary revascularization by CABG on‐ and off‐pump and PCI with bare‐metal stents (BMSs), DESs, or percutaneous transluminal coronary angioplasty (PTCA) to determine clinical outcomes.

**Methods and Results:**

All patients undergoing isolated coronary revascularization in 8 community‐based hospitals were enrolled. Final follow‐up was obtained after 5 years by patient and/or physician contact and the Social Security Death Index. ST‐elevation myocardial infarction and salvage patients were excluded. Five or more years of follow‐up was obtained on 81.5% (3156) of the eligible patients—968 CABG patients (82.0%) and 2188 PCI patients (81.3%). Overall follow‐up was 63.5±27.9 months (median**,** 79.7 months). The incidence of initial major adverse cardiac events (MACEs) at follow‐up for CABG versus PCI was 29.2% versus 41.8% (*P*<0.001). Analysis of stent subgroups showed more events with BMSs (equivalent to PTCA alone) compared with DESs. All stents had more events than on‐ or off‐pump CABG groups. Using propensity score–matched groups, the odds ratio for CABG to PCI was 0.69 (95% confidence interval [CI], 0.56 to 0.85; *P*<0.001) for mortality and 0.58 (95% CI, 0.45 to 0.75; *P*<0.001) for any MACE.

**Conclusions:**

In the current era of DES and off‐pump surgery, in a community hospital setting, comparable patients undergoing coronary revascularization appear to benefit from improved long‐term survival and reduced MACE with CABG versus PCI.

## Introduction

With the improvements in percutaneous coronary intervention (PCI) techniques and the development of drug‐eluting stents (DESs), revascularization for coronary artery disease has shifted from coronary artery bypass grafting (CABG) to PCI procedures. Today >75% of revascularization cases are performed using PCI.^1^ In the Coronary Artery Revascularization Evaluation (CARE) study reported here and previously,^2^ 71.2% of the patients were treated using PCI. Current approaches are segmented into on‐pump and off‐pump CABG, PCI using bare‐metal stents (BMSs) or DESs, and patients receiving only percutaneous transluminal coronary angioplasty (PTCA). Selection of the proper revascularization strategy for an individual patient is based on several factors including the clinician's knowledge of expected outcomes and personal clinical experience. In turn, much of clinician knowledge of expected procedural outcomes comes from randomized controlled trials (RCTs) comparing approaches.^3–6^

RCTs are usually considered to represent the highest level of evidence‐based medicine, but their results must be kept in perspective. In these trials, the selection criteria may produce a design bias, with a study group that may not be representative of the actual population being treated outside the study. Generally between 3% and 8% of patients screened for inclusion in randomized trials comparing CABG versus PCI have actually been enrolled in these trials.^7–10^

Taking the observed results and extrapolating them to the clinical population being treated may be problematic, especially because there are reports of >70% off‐label use of DESs.^11–12^ The validity of this “generalization” of the results in these select patients to the population as a whole is questionable. Similar issues exist for randomized trials of CABG procedures. Doubts about the correlation of RCT findings with the “real‐world” results of coronary revascularization by either PCI or CABG have been raised by analyses of large population outcomes databases.^13–14^

We hypothesized that the results of coronary revascularization as performed in routine clinical practice might differ from the outcomes of randomized trials. The CARE study was designed to reflect real‐world practice, a registry of procedures and outcomes from 8 community‐based practices.

## Methods

As previously published,^2^ this was a multicenter registry recording patient data and procedures from 8 centers. There were no patient selection criteria specified, and the physicians were free to select and treat according to their preferences. Overall, catheter‐based procedures were used on 71.2% of the patients (3090 of 4338), who made up the PCI group, with individual centers varying between 54% and 80% in their usage. The comparison group consisted of patients having a CABG procedure either on‐ or off‐pump (the CABG group). Data were collected locally and returned to a central repository, where they were entered into a proprietary database. Data were collected over a 6‐month period from February 1, 2004, until July 31, 2004. All collected data were subjected to quality checks and data validation tests searching for inconsistencies and errors. Those patient records and ones containing missing data were referred back to the participating center for correction or completion. Returned patient records were updated in the master database.

A final follow‐up was scheduled to begin 5 years after date of procedure. This process continued until summer 2011, when the database was locked. Follow‐up included Social Security Death Index (SSDI) searches, hospital and physician inquiries, and letters and telephone calls to the patients. The time lapse between surgery and the final follow‐up made contacting patients difficult, accounting for the prolonged period spent locating patients. SSDI searches were carried out on all the initial patients in the study.

Data were extracted from the database, and the initial data set was combined with the follow‐up data. Patients with a diagnosis of ST‐elevation myocardial infarction (STEMI) or status listed as salvage were deleted from the data set for analysis. Data were summarized and compared using chi‐square statistics for discrete variables and *t* tests or analysis of variance for continuous data. The propensity matching used 18 preoperative risk parameters and accounted for nesting of patients in hospitals. The scores were put through a 1‐to‐1 greedy matching algorithm, which produced 2 equivalent data sets for PCI and CABG patients with 5+ years of follow‐up.

Survival curves were derived using Kaplan–Meier methods, whereas hazard ratios and cumulative hazards were calculated using the Cox proportional hazard model. Odds ratios were determined using hierarchical logistic regression (SAS Genmod procedure). All statistical calculations used SAS 9.3 (SAS Institute, Cary, NC).

The protocol was approved by the Institutional Review Board for North Texas at Medical City Dallas Hospital with a waiver of consent.

## Results

The initial study collected treatment data on 4338 patients (1248 with CABG and 3090 with a PCI intervention). After eliminating STEMI and salvage patients, there were 3871 patients (1181 CABG and 2690 PCI). Five‐plus years of follow‐up was obtained for 81.5% of the eligible patients (3156)—968 CABG patients (82.0%) and 2188 PCI patients (81.3%). Mortality data from the SSDI searches were collected on all the original 4338 patients.

The follow‐up period was slightly longer for the CABG group at 67.2±25.8 months (median, 84 months); it was 61.8±28.6 months (median, 79.4 months) for the PCI group (*P*<0.001). To ensure that the patients who were dropped from analysis because they could not be followed did not bias the data set, we compared the properties of those with follow‐up with the initial patient set. None of the measured parameters differed significantly either statistically or clinically except for patient age (as reported at the time of the procedure), with a slight trending to 1.5 years older in the follow‐up group (Table S1).

**Figure 1.**
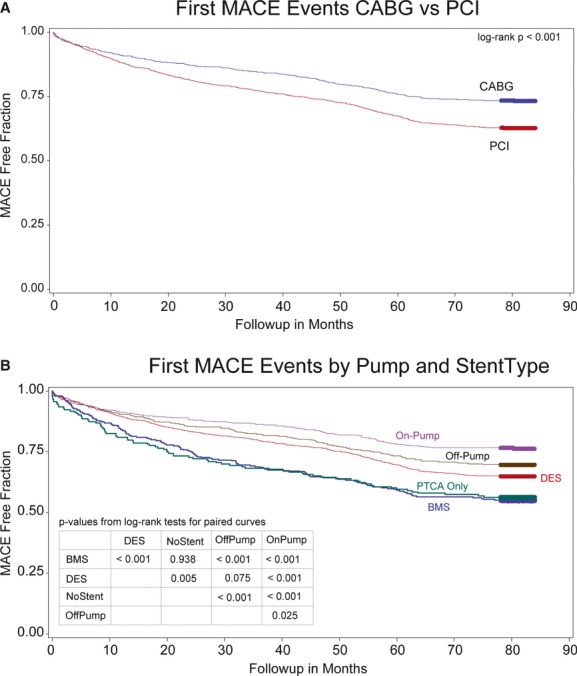
Kaplan–Meier curve for freedom from MACE stratified into CABG and PCI groups (A) and MACE stratified by treatment group (B). MACE indicates major adverse cardiac event; CABG, coronary artery bypass grafting; PCI, percutaneous coronary intervention.

**Figure 2.**
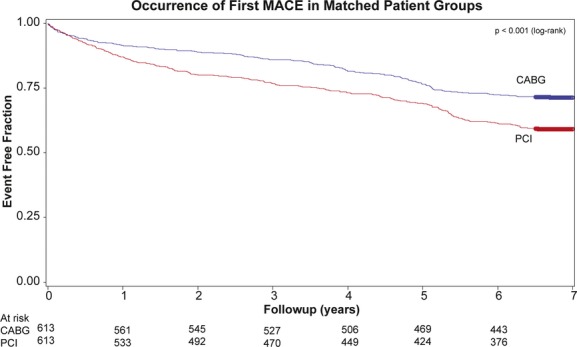
Comparison of MACE‐free period in propensity score–matched CABG and PCI patient groups. MACE indicates major adverse cardiac event; CABG, coronary artery bypass grafting; PCI, percutaneous coronary intervention.

**Figure 3.**
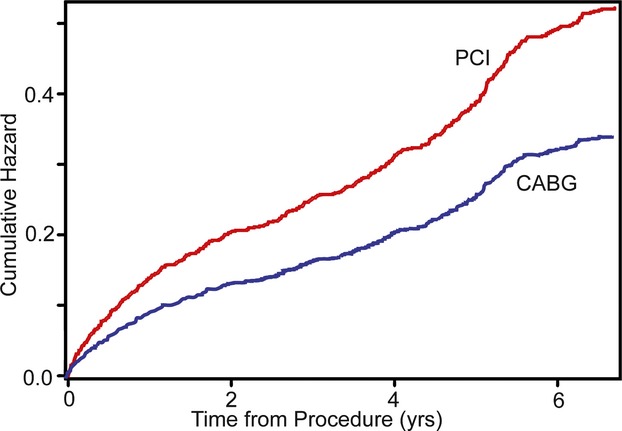
Accumulation of MACEs in matched patient groups. MACE indicates major adverse cardiac event; CABG, coronary artery bypass grafting; PCI, percutaneous coronary intervention.

There were numerous differences between the PCI and CABG groups. The PCI group had a higher proportion of previous PCI, previous CABG, elective status, cerebrovascular disease, dyslipidemia, and single‐ and double‐vessel disease. The CABG group, on the other hand, had a higher proportion of prior stroke, hypertension, prior MI, arrhythmia, urgent status, peripheral vascular disease, triple‐vessel disease, and reduced ejection fraction (Table 1). The follow‐up data from these groups were analyzed to determine the first occurrence of a major cardiac adverse event (MACE) for a given patient, both the type and the time from initial procedure. Overall, 41.8% of the PCI group and 29.2% of the CABG group had a MACE (*P*<0.001). Each of the recorded events was more frequent in the PCI than in the CABG group (Table 2). Moreover, there is an apparent hierarchy of mortality and revascularization favoring on‐pump versus off‐pump CABG, and DES versus BMS and PTCA alone (Table 3, Figure 1). Because risk factors varied significantly between the CABG and PCI groups, which were selected by physician clinical preference (Table 1), propensity score matching was used to create 2 equivalent groups of follow‐up patients. The match yielded 613 patients in each group with no statistical or clinically significant differences between groups except that the CABG group had an approximately 10% lower mean ejection fraction at the time of treatment (Table S2). The 613 CABG patients had a total of 175 MACEs (28.5%), whereas the 613 patients treated by PCI initially had 250 events (40.8%), *P*<0.001 (Table 4). The mean time to first event in the groups was calculated as 34±24 months for CABG and 33±25 months for PCI (*P*=0.476). Examining the median values (39 months for CABG and 28 months for PCI) shows a skew to earlier events in the PCI group (Figure 2). The odds ratio (OR) for mortality comparing CABG to PCI was 0.69 (95% CI, 0.56 to 0.85; *P*<0.001), whereas the odds ratio for any MACE was 0.58 (95% CI, 0.45 to 0.75; *P*<0.001; Table 5). This trend is also reflected in the cumulative hazards curve in Figure 3. Of note, there was no difference in the preoperative risk (the Society of Thoracic Surgeons (STS) predicted risk of mortality: for off‐pump CABG, 2.20±3.10; for on‐pump CABG, 2.37±2.77; *P*=0.392) or perioperative mortality (off‐pump, 2.2% (10/453); versus on‐pump, 2.1% (11/515); *P*=0.828) between the off‐pump and on‐pump CABG patients.

**Table 1.** Comparison of Demographics Between Patients Originally Having a CABG or PCI Procedure

Variable	CABG	PCI	*P* Value
Number of patients	973	2255	
Men	69.7% (677/972)	68.2% (1536/2253)	0.408
Preoperative stroke	8.0% (78/973)	4.7% (106/2250)	<0.001
Previous CV intervention	22.7% (220/971)	48.1% (1085/2254)	<0.001
Previous CABG surgery	5.6% (54/971)	22.8% (515/2254)	<0.001
Previous valve surgery	0.1% (1/971)	0.6% (13/2251)	0.060
Previous PCI procedure	18.6% (181/971)	35.2% (791/2250)	<0.001
Hypertensive	79.2% (771/973)	76.0% (1713/2254)	0.045
Angina	86.7% (841/970)	85.5% (1915/2240)	0.366
Heart failure	8.2% (79/969)	8.1% (181/2247)	0.926
Renal failure	5.2% (51/973)	5.0% (113/2254)	0.786
On dialysis	1.5% (15/972)	1.6% (36/2238)	0.892
Preoperative MI	31.1% (298/958)	27.6% (621/2254)	0.041
Arrhythmia	7.7% (75/970)	1.8% (39/2155)	<0.001
Diabetes	34.4% (335/973)	33.9% (763/2254)	0.750
On insulin	11.0% (107/973)	11.1% (250/2255)	0.941
Triple‐vessel disease	63.2% (554/877)	20.4% (401/1968)	<0.001
Single‐vessel disease	8.6% (75/877)	47.6% (937/1968)	<0.001
Two‐vessel disease	28.3% (248/877)	32.0% (630/1968)	0.046
Preoperative inotrope use	0.8% (8/963)	1.4% (32/2225)	0.157
Elective status	44.1% (426/965)	75.1% (1648/2193)	<0.001
Family history	49.1% (478/973)	46.9% (1056/2254)	0.235
Cerebrovascular disease	11.6% (113/973)	19.4% (437/2254)	<0.001
Peripheral arterial disease	12.5% (122/973)	9.9% (222/2253)	0.023
Current/recent smoker	52.7% (512/972)	44.5% (1002/2254)	<0.001
Dyslipidemia	57.0% (555/973)	70.6% (1592/2254)	<0.001
Preoperative beta blockade	63.6% (612/963)	51.6% (1147/2224)	<0.001
Age, y	64.80±10.36	65.32±11.90	0.217
Ejection fraction	50.08±11.90	53.78±16.81	<0.001

CABG indicates coronary artery bypass grafting; PCI, percutaneous coronary intervention; CV, cardiovascular; MI, myocardial infarction.

**Table 2.** First Events in CABG and PCI Groups

Event	CABG (n=968)	PCI (n=2188)	*P* Value
Mortality*	198 (16.8%)	544 (20.2%)	0.007
CABG	6 (0.6%)	67 (3.1%)	<0.001
MI	21 (2.2%)	67 (3.1%)	0.160
PTCA	3 (0.3%)	31 (1.4%)	0.006
Stent	55 (5.7%)	206 (9.4%)	<0.001

CABG indicates coronary artery bypass grafting; PCI, percutaneous coronary intervention; MI, myocardial infarction; PTCA, percutaneous transluminal coronary angioplasty; SSDI, Social Security Death Index.

* Mortality follow‐up was 100% using SSDI. Percent value for mortality was calculated using all eligible patients.

**Table 3.** First Events Separated by Treatment Modality

	BMS (n=305)	DES (n=1685)	PTCA (n=198)	Off‐Pump (n=453)	On‐Pump (n=515)
Mortality*	96 (25.6%)	396 (19.1%)	52 (21.7%)	109 (19.3%)	89 (14.4%)
CABG	6 (2.0%)	47 (2.8%)	14 (7.1%)	4 (0.9%)	2 (0.4%)
MI	5 (1.6%)	56 (3.3%)	6 (3.0%)	7 (1.6%)	14 (2.7%)
PTCA	7 (2.3%)	19 (1.1%)	5 (2.5%)	2 (0.4%)	1 (0.2%)
Stent	32 (10.5%)	156 (9.3%)	18 (9.1%)	25 (5.5%)	30 (5.8%)

BMS indicates bare‐metal stent; DES, drug‐eluting stent; PTCA, percutaneous transluminal coronary angioplasty; CABG, coronary artery bypass grafting; MI, myocardial infarction; SSDI, Social Security Death Index.

* Mortality follow‐up was 100% using SSDI. Percent value for mortality was calculated using all eligible patients.

**Table 4.** First MACE in Matched Treatment Groups

Event	CABG	PCI	*P* Value
Mortality	22.68% (139)	31.65% (194)	<0.001
CABG	0.49% (3)	1.63% (10)	0.051
MI	1.63% (10)	1.96% (12)	0.667
PTCA	0.16% (1)	1.47% (9)	0.011
Stent	3.59% (22)	4.08% (25)	0.655
Any revascularization (CABG, PTCA, stent)	4.24% (26)	7.18% (44)	0.027
Any MACE	28.55% (175)	40.78% (250)	<0.001
No MACE	71.45% (438)	59.22% (363)	<0.001

MACE indicates major adverse cardiac event; CABG, coronary artery bypass grafting; PCI, percutaneous coronary intervention; MI, myocardial infarction; PTCA, percutaneous transluminal coronary angioplasty.

**Table 5.** Odds Ratio for Individual MACE in Matched Treatment Groups

Event	Odds Ratio (CABG vs PCI) (95% Confidence Interval)	*P* Value
Mortality	0.69 (0.56 to 0.85)	<0.001
CABG	0.25 (0.14 to 0.44)	<0.001
MI	0.83 (0.51 to 1.33)	0.432
PTCA	0.24 (0.08 to 0.75)	0.014
Stent	0.69 (0.55 to 0.86)	0.001
Any revascularization (CABG, PTCA, stent)	0.55 (0.34 to 0.91)	0.020
Any MACE	0.58 (0.45 to 0.75)	<0.001

MACE indicates major adverse cardiac event; CABG, coronary artery bypass grafting; PCI, percutaneous coronary intervention; MI, myocardial infarction; PTCA, percutaneous transluminal coronary angioplasty.

## Discussion

With the overall improvements in coronary stents, and the introduction of drug‐eluting stents, interventional cardiologists and their patients are more often choosing PCI as the initial treatment strategy for coronary revascularization. This has led to multiple studies comparing outcomes of patients treated using PCI or CABG surgery. It is generally held that the RCT is the ”gold standard” because randomization avoids selection bias as well as minimizing the influence of unrecognized confounding variables. Although RCTs tend to have high internal validity, the formal methodology and criteria make their external validity or generalizability questionable. The study population of the RCT may be far from that seen in routine clinical practice, making transferring study conclusions to the more general clinical practice population harder to justify. Observational studies may lack some internal validity (although patient‐matching procedures can minimize this) but may more closely reflect the outcomes seen in clinical practice.

Although some RCTs comparing CABG with PCI have shown no differences in mortality between the 2 treatment strategies, virtually all have found an increased need for repeat revascularization in patients treated by PCI. A meta‐analyses of 13 RCTs showed that in multivessel disease CABG had a significant advantage over PTCA at 5 and 8 years in survival and reduced the need for revascularization, emphasizing the need for long‐term follow‐up.^15^ A more recent meta‐analysis of RCTs from the stent era demonstrated no difference in events rates of death, stroke, or MI at 5 years, but a higher incidence of repeat revascularization.^16^ Although early results of the SYNTAX trial demonstrated no difference in mortality,^17^ the most recent 5‐year follow‐up reports a highly significant difference in all‐cause death (9.2% versus 14.6%; *P*=0.006) as well as cardiac death (4.0% versus 9.2%; *P*<0.001), favoring CABG versus PCI in for patients with triple‐vessel disease. As anticipated, repeat revascularization also favored CABG (12.6% versus 25.4%; *P*<0.001). Results for patients with left main disease, however, showed equivalent mortality (14.6% versus 12.8%; *P*=0.53) but lower revascularization (15.5% versus 26.7%; *P*<0.001) with CABG versus PCI (http://www.theheart.org/article/1466345/print.do; accessed November 22, 2012).

Although results have varied depending on the risk profile of the study population, large retrospective registry data appears to favor CABG over PCI for patients with multivessel disease. Looking at matched pairs of patients in long‐term follow‐up of patients in the New York State database showed an overall 8‐year survival of 78.0% for CABG and 71.2% for stenting (HR, 0.68; 95% CI, 0.64 to 9.74; *P*<0.001).^18^ The recently published ASCERT trial (observational with an inverse propensity weighting) combined records from the Society of Thoracic Surgeons' database, the American College of Cardiology Foundation database, and Centers for Medicare and Medicaid Services (CMS) records to trace events and survival for 189 793 patients undergoing CABG or PCI. After a mean follow‐up of 2.72 years (median, 2.67 years), they reported Kaplan–Meier survival at 4 years for PCI that was only 79% of the survival measured for CABG. CABG had a survival advantage from the 1 year point onward.^19^

In our observational study, PCI compared with CABG had a higher mortality rate (20.2% versus 16.8%) and a higher rate of target vessel revascularization (12.9% versus 6.6%). To reduce biases between groups, propensity score–matched subgroups were created to eliminate the differences in risk factors and demographics seen between the unmatched groups. In these matched groups, mortality was higher in the PCI group versus the CABG group (31.7% versus 22.7%), whereas PCI‐treated patients had a higher rate of target vessel revascularization (7.2% versus 4.2%) than CABG‐treated patients. This study shows that over the nearly 7‐year mean follow‐up, the patients initially treated with PCI had higher rates of mortality, MI, and target‐vessel revascularization. All the curves showed divergence over time, emphasizing the importance of longer term follow‐up in elucidating differences between treatment groups.

The observed difference in mortality between DES and BMS patients was somewhat unexpected. A recent meta‐analysis of 76 RCTs comparing DES with BMS in 57 138 patients demonstrated a 39% to 61% reduction in target‐vessel revascularization with varying types of DES, but no difference in mortality.^20^ Available data did not permit us to assess for appropriateness of stent selection, and consensus in this regard has changed since the study period. Mortality differences may therefore reflect differences in patient selection rather than stent selection per se. With reports of off‐label usage of DESs in the 70% range,^11–12^ these data are more reflective of clinical practice rather than the more defined patient selection criteria that would be anticipated in the context of RCTs. Similarly, the relatively lower revascularization rates observed here may reflect a more aggressive initial strategy, with stenting of less severe lesions or less complete continuity of patient care than we have come to expect from the RCTs. Unfortunately, we do not have any data available regarding SYNTAX scoring of lesions, as the study period preceded the release of this methodology.

The improved late survival of patients with multivessel disease undergoing PCI versus either on‐pump or off‐pump surgery has been previously reported.^21^ Even though there is solid evidence of comparable perioperative mortality rates with on‐ versus off‐pump CABG,^22^ the higher mortality for off‐pump versus on‐pump surgery at follow‐up has also been previously reported in a meta‐analysis of RCTs.^23^ Certainly in this clinical registry, there may well have been variations in operator skill with an emerging technique, perhaps resulting in variability in long‐term graft patency.^24^

### Limitations

The major limitation to this cross‐sectional follow‐up study is that when patients were found to have died (in an SSDI search), their families were usually not contacted further. As a result, we are unaware of an earlier MACE, and so events other than death may be underreported. Some data were available from physician offices, but the available data may not have reflected complete patient history. This limitation may have partially accounted for the relatively low revascularization rates recorded.

As a retrospective multicenter observational study, patient groups may reflect multiple potential treatment biases. Although this report includes the results from an analysis of propensity‐score matched CABG and PCI patient groups, which were well matched for patient variables, there may still be factors not accounted for in the models that may have affected outcome.

Follow‐up was achieved in just over 80% of the study population, using a variety of methods. A highly mobile population combined with changing phone numbers and replacement of landlines with cellular phones prevented a higher percentage of follow‐up. There is no a priori reason to believe that this limitation would bias 1 group over the other. However, comparison of 30 variables in the patient group in whom follow‐up was achieved with those from the entire patient cohort revealed no significant difference in any parameter except for a slightly older average age in the patients contacted. Moreover, with the use of the SSDI, mortality data were 100% complete.

Finally, we are unable to assess the stroke rate on these cases because only periprocedural data are available. Our initial study reported a relatively low incidence of periprocedural stroke (0.1% PCI versus 0.7% CABG; *P*=0.003); however, long‐term risk could not be evaluated. Recent evidence suggests that this may represent an important element to include in future investigations.^25^

## Conclusion

In conclusion, in the current era of DES and off‐pump surgery, in a community hospital setting, comparable patients undergoing coronary revascularization appear to benefit from improved long‐term survival and reduced MACEs with CABG versus PCI. Results emphasize the need to evaluate therapeutic options within the context of real‐world clinical practice, as well as the importance of long‐term follow‐up.
